# Intraprocedural atrial flutter transition predicts arrhythmia-free survival after “2C3L Plus” ablation for long-standing persistent atrial fibrillation

**DOI:** 10.3389/fcvm.2026.1781094

**Published:** 2026-04-24

**Authors:** Tong Liu, Kangning Han, Yang Yang, Deyong Long

**Affiliations:** Department of Cardiology, Beijing Anzhen Hospital, Capital Medical University, Beijing Institute of Heart, Lung and Blood Vessel Diseases, Beijing, China

**Keywords:** 2C3L plus, atrial fibrillation termination, atrial flutter, long-standing persistent atrial fibrillation, recurrence

## Abstract

**Background:**

Long-standing persistent atrial fibrillation (LSPAF) remains a challenge of catheter ablation. The efficiency and optimal procedural endpoints of “2C3L plus” approach—a strategy combining pulmonary vein isolation (PVI) with linear and complex fractionated atrial electrogram (CFAEs) ablation—is unclear.

**Methods:**

This single-center, retrospective cohort study included 260 consecutive patients with LSPAF (defined as continuous AF lasting > 12 months) who underwent *de novo* radiofrequency catheter ablation between January 2020 and January 2022. All patients received a standardized “2C3L plus” strategy. The primary endpoint was freedom from any documented atrial tachyarrhythmia lasting >30 s, off antiarrhythmic drugs, at 1-year follow-up. Predictors of recurrence were analyzed using multivariable Cox regression analysis.

**Results:**

Intraprocedural atrial fibrillation (AF) termination was achieved in 103 of 260 (39.6%) patients and 90 (34.6%) patients converted to atrial flutter (AFL) during ablation. Acute termination of AF directly to sinus rhythm (SR) was not associated with a lower risk of recurrence (adjusted HR: 0.765, 95% CI: 0.410–1.428, *P* = 0.400). However, intraprocedural conversion from AF to AFL was associated with significantly reduced recurrence risk (Uni: HR: 0.319, 95% CI: 0.142–0.714, *P* = 0.005; adjusted HR: 0.306, 95% CI: 0.133–0.704, *P* = 0.005). Further analysis revealed that the sequential intraprocedural conversion of AF-AFL-SR during ablation was a strong and independent predictor of arrhythmia-free survival (Uni: HR: 0.275, 95% CI: 0.108–0.696, *P* = 0.006; adjusted HR: 0.305, 95% CI: 0.119–0.784, *P* = 0.014).

**Conclusion:**

In patients with LSPAF undergoing extensive “2C3L Plus” substrate ablation, the intraprocedural organization of AF-AFL-SR, rather than AF termination itself, emerged as a powerful independent predictor of 1-year arrhythmia-free survival, suggesting its value as a more meaningful prognostic endpoint.

## 1 Introduction

Atrial fibrillation (AF) is the most common sustained cardiac arrhythmia, and its natural course often progresses from paroxysmal to persistent, ultimately evolving into long-standing persistent AF (LSPAF) lasting over one year. This progression is accompanied by significant atrial electrical and structural remodeling, creating a complex substrate that sustains the arrhythmia and renders LSPAF particularly challenging to treat (1).

Catheter ablation has become a cornerstone therapy for restoring and maintaining sinus rhythm. Pulmonary vein isolation (PVI), which electrically isolates the pulmonary veins as the primary trigger for AF, is universally recognized as the fundamental element of all AF ablation procedures. However, for LSPAF patients, due to extensive underlying atrial remodeling, PVI alone yields limited success. This has driven clinical exploration of strategies combining PVI with additional substrate modification. These strategies primarily include two approaches: 1) creating linear ablation lines in the left atrium to segment the atria and block macro-reentrant circuits; and 2) ablating complex fractionated atrial electrograms (CFAEs), which are considered key drivers perpetuating AF.

In this context, our center previously proposed the “2C3L” ablation strategy, combining CPVI (“2C”) with three linear ablation lines (“3L”): the left atrial roof line, mitral isthmus line, and tricuspid isthmus line. The recent PROMPT-AF trial, which tested Ethanol Infusion of the Vein of Marshall (EIVOM)-assisted “2C3L” strategy in persistent AF, reported a significantly higher 1-year success rate of 70.7% than PVI alone (2). Given the even more complex substrate in LSPAF, we further developed the “2C3L plus” strategy by adding extensive complex fractionated atrial electrograms (CFAEs) ablation to “2C3L”, aiming to achieve intraprocedural AF termination. However, this “more-is-better” philosophy of extensive substrate modification faced significant challenges from subsequent large randomized clinical trials. The landmark STAR-AF II trial demonstrated that for persistent AF, adding either CFAEs ablation or linear ablation to PVI provided no additional benefit (3). The RASTA trial even found that adding CFAEs ablation resulted in worse outcomes (4). Furthermore, the prognostic value of intraprocedural AF termination—a key procedural endpoint of extensive substrate modification—has become highly debated. A subgroup analysis of STAR-AF II indicated that although additional substrate modification significantly increased the intraprocedural AF termination rate, AF termination itself did not predict long-term success (5).

Given this background and controversies, this study aims to retrospectively analyze the outcomes of the “2C3L plus” strategy in LSPAF patients. Our primary objectives are: 1) to describe the one-year freedom from AF recurrence off antiarrhythmic drugs (AADs) and the intraprocedural AF termination rate in LSPAF patients treated with this strategy; 2) to re-evaluate whether intraprocedural AF termination predicts AF recurrence specifically under this strategy; and 3) to explore the relationship between different modes of AF termination (direct conversion to sinus rhythm vs. conversion through AFL/AT transitional rhythm.

## 2 Materials and methods

This was a single-center, retrospective observational study. A total of 260 inpatients with LSPAF who underwent *de novo* radiofrequency ablation were consecutively enrolled in this study from January 2020 to January 2022 at Anzhen Hospital. LSPAF was defined as continuous atrial fibrillation (AF) persisting for more than 12 months, despite attempts to restore sinus rhythm. The major exclusion criteria were paroxysmal atrial fibrillation, history of AF ablation, AF lasting less than 12 months, rheumatic heart disease, and loss to follow-up. This study was performed following the Helsinki Declaration of Human Rights and was approved by the institutional review board of Beijing Anzhen Hospital, Capital Medical University (Ethics Number: 2026033X).

Data regarding the demographic characteristics, clinical features and laboratory examinations were collected for all subjects including sex, age, past medical history (hypertension, diabetes mellitus), smoking status, body mass index, blood pressure, heart rate (HR), systolic blood pressure (SBP), left atrial diameter (LAD), left ventricular ejection fraction (LVEF), and albumin.

Before ablation, oral anticoagulation was given for at least four weeks. Transesophageal echocardiography or intracardiac echocardiography was performed to exclude left atrial thrombus. During RFCA, heparin was administered to maintain an activated clotting time of more than 300 s. After RFCA, anti-arrhythmic drugs and oral anticoagulation were given for at least three months. Under a CARTO mapping system (CARTO; Biosense Webster, Inc., Irvine, California), RFCA was performed at a maximum temperature of 45 °C, maximum power of 50 W, and flow rate of ≥15 mL/min. Ablation index targets will be set with 500–550 for anterior wall; 350–400 for posterior wall; 450–550 for the LA roof and CTI; 550–600 for MI. First, the CPVI, roofline, mitral isthmus, and cavotricuspid isthmus were ablated one by one. Then, the bottom-line, left atrial CFAE and right atrial CFAE were ablated one by one. However, if termination of AF occurred during this linear ablation phase, the following CFAE ablation steps were not performed. All CFAEs defined as electrograms with continuous activity or complex fractionated electrogram mean detected by the system of <80 ms were eliminated.

Whenever AF organized into atrial flutter (AFL) documented by a sudden change to regular tachycardia with fixed cycle length and consistent activation pattern, detailed activation mapping was immediately performed. The re-entry circuit was mapped with high-density multipolar catheters. Targeted RF applications were delivered at the critical isthmus until complete conduction block across the circuit was achieved and AFL terminated. If AFL could not be terminated after meticulous mapping and ablation, or if AF persisted without transitioning to AFL, external direct-current cardioversion was performed under deep intravenous sedation to restore sinus rhythm.

Linear block are validated under sinus rhythm or atrial pacing: Roofline bidirectional block will be confirmed by: 1) caudal-cranial activation pattern in the posterior wall under sinus rhythm or pacing anterior to the ablation line (LAA) and 2) caudal-cranial activation in the anterior wall under pacing posterior to the ablation line. MI bidirectional block is confirmed by: 1) proximal-to-distal CS activation pattern when pacing at the LAA and left lateral ridge; 2) the activation of the anterior mitral annulus is from the septum to the LA lateral wall and then to ablation line when pacing at the distal CS. CTI bidirectional block is confirmed by: 1) the activation is from the septum to the ablation line when pacing at RA free wall; 2) the activation is from the RA lateral wall to the ablation line when pacing at the proximal CS.

Patients were followed-up by telephone and outpatient clinic visit. Twenty-four-hour Holter monitoring was checked at 1, 2, 3, 6, 9 and 12 months after discharge. In addition, if patients felt serious symptoms of arrhythmia, they were required to undergo an electrocardiogram at the closest hospital. AF recurrence was defined as documented atrial tachycardia, atrial flutter or AF for at least 30 s during the 1-year follow-up. The final diagnosis of recurrence was reviewed by two cardiologists.

The statistical computations were performed using R version 4.5.0 (The R Project for Statistical Computing, Vienna, Austria). Continuous variables are reported as the means ± standard deviations for normally distributed data or medians and interquartile for non-normally distributed data. They were compared using Student's *t*-test if normally distributed or the Mann–Whitney *U*-test if nonnormally distributed. We used the Kolmogorov‒Smirnov test to check their normality. Discrete variables are expressed as frequencies and percentages and were compared using the chi-square test. Multivariable Cox regression analysis was performed to detect the independent risk factor for AF recurrence with the adjustment of age, sex, smoking, drinking, LAD, history of hypertension, diabetes and LVEF. The Kaplan–Meier method was used to estimate the relationship between freedom from atrial tachyarrhythmias and ablation termination or AFL. A two-sided *p* value <0.05 was considered statistically significant.

## 3 Results

Baseline characteristics of the participants are summarized in Table 1. The average age was 55.8 ± 7.3 years, and 195 (75.0%) patients were male. Patients in the recurrence group had a significantly larger LAD compared to those in the no-recurrence group. There were no significant differences in the incidence of hypertension, diabetes, severe mitral regurgitation, or severe tricuspid regurgitation between the two groups (all *P* > 0.05).

**Table 1:** Baseline characteristics of the recurrence and no recurrence.

Variables	All	Recurrence	No recurrence	*P* Value
	*n* = 260	*n* = 45	*n* = 215	
Male, *n* (%)	195 (75.0)	37 (82.2)	158 (73.5)	0.219
Age, years	55.8 ± 7.3	56.4 ± 8.5	55.6 ± 7.0	0.511
AF duration, month	51.6 ± 47.0	71.9 ± 49.7	47.4 ± 45.4	<0.001
Left atrial diameter, mm	42.8 ± 4.2	46.6 ± 4.7	42.0 ± 43.6	<0.001
LVEF, %	51.8 ± 3.3	51.8 ± 3.5	51.8 ± 3.2	0.892
BMI, Kg/m^2^	25.4 ± 1.6	26.3 ± 2.0	25.2 ± 1.4	<0.001
Severe mitral valve regurgitation, *n* (%)	27 (10.4)	8 (17.8)	19 (8.8)	0.074
Severe tricuspid valve regurgitation, *n* (%)	36 (13.8)	12 (26.7)	24 (11.2)	0.066
Hypertension, *n* (%)	124 (47.7)	26 (57.8)	98 (45.6)	0.136
Diabetes mellitus, *n* (%)	82 (31.5)	17 (37.8)	65 (30.2)	0.322
Systolic blood pressure, mmHg	127.6 ± 8.0	128.2 ± 10.1	127.4 ± 7.5	0.560
Diastolic blood pressure, mmHg	79.6 ± 6.6	80.1 ± 6.8	79.5 ± 6.6	0.550
Fasting plasma glucose, mmol/L	5.46 ± 1.03	5.91 ± 0.87	5.36 ± 1.04	0.001
Smoking, *n* (%)	95 (36.5)	14 (31.1)	81 (37.7)	0.406
Alcohol consumption, *n* (%)	76 (29.2)	18 (40.0)	58 (27.0)	0.081
PVI ablation, *n* (%)	260 (100)	45 (100)	215 (100)	-
Roofline ablation, *n* (%)	260 (100)	45 (100)	215 (100)	-
Mitral isthmus ablation, *n* (%)	260 (100)	45 (100)	215 (100)	-
Mitral isthmus block achieved, *n* (%)	230 (88.5)	39 (86.7)	191 (88.8)	0.679
Cavotricuspid isthmus ablation, *n* (%)	260 (100)	45 (100)	215 (100)	-
Cavotricuspid isthmus block achieved, *n* (%)	252 (96.9)	44 (97.8)	208 (96.7)	0.715
Bottom line, *n* (%)	237 (91.2)	37 (82.2)	200 (93.0)	0.119
Left atrial CFAE, *n* (%)	222 (85.4)	41 (91.1)	181 (84.2)	0.232
Right atrial CFAE, *n* (%)	195 (75.0)	36 (80.0)	159 (74.0)	0.394
Superior vena cava, *n* (%)	42 (16.2)	8 (17.8)	34 (15.8)	0.745
Coronary sinus, *n* (%)	125 (48.1)	24 (53.3)	101 (47.0)	0.438
Ethanol ablation of the vein of Marshall, *n* (%)	37 (14.2)	5 (11.1)	32 (14.9)	0.510
Switched to AFL, *n* (%)	90 (34.6)	7 (15.6)	83 (38.6)	0.003
AFL ablation termination, *n* (%)	76 (29.2)	5 (11.1)	71 (33.0)	0.003
Terminate AF, *n* (%)	103(39.6)	15(33.3)	88(40.9)	0.404

AF, atrial fibrillation; LVEF, left ventricular ejection fractions; BMI, body mass index; PVI, pulmonary vein isolation; CFAE, complex fractionated atrial electrograms; AFL, atrial flutter.

Intraprocedural AF termination by ablation was achieved in 103 patients (39.6%). During ablation, 90 patients (34.6%) converted to AFL; of these, 38 (42.2%) occurred AFL after completion of the bottom line, 27 (30.0%) after left atrial CFAE ablation, 15 (16.6%) after right atrial CFAE ablation, 7 (7.8%) after MI line ablation and 3 (3.3%) after PVI ablation. Subsequent mapping during AFL confirmed MI-dependent AFL in 42 (46.7%), CTI-dependent AFL in 30 (33.3%), roofline-dependent AFL in 7 (7.8%), and other forms of AFL in 11 (12.2%) (Table 2). 76 were subsequently converted to sinus rhythm with further ablation, while 14 required cardioversion (CV). A comparison of clinical and procedural characteristics between patients who achieved AF termination via ablation (termination group, *n* = 103) and those who required CV (CV group, *n* = 157) is presented in Table 2. Patients in the termination group had a significantly smaller LAD and shorter AF duration compared to the CV group.

**Table 2:** Clinical and procedural characteristics of patients.

Variables	Termination	Cardioversion	*P* Value
	*n* = 103	*n* = 157	
Male, *n* (%)	80 (77.7)	115 (73.2)	0.421
Age, years	55.4 ± 6.5	56.0 ± 7.8	0.516
AF duration, month	37.8 ± 26.8	60.7 ± 54.6	0.004
Left atrial diameter, mm	41.6 ± 3.9	43.6 ± 4.2	<0.001
LVEF, %	51.8 ± 3.2	51.8 ± 3.4	0.989
BMI, Kg/m^2^	25.2 ± 1.3	25.5 ± 1.7	0.205
Severe mitral regurgitation, *n* (%)	12 (11.7)	15 (9.6)	0.588
Severe tricuspid regurgitation, *n* (%)	15 (14.6)	21 (13.4)	0.786
Hypertension, *n* (%)	43 (41.7)	81 (51.6)	0.120
Diabetes mellitus, *n* (%)	34 (33.0)	48 (30.6)	0.679
Systolic blood pressure, mmHg	128.4 ± 8.4	127.1 ± 7.6	0.194
Diastolic blood pressure, mmHg	79.4 ± 25.9	79.7 ± 7.1	0.676
Fasting plasma glucose, mmol/L	5.60 ± 1.05	5.36 ± 1.00	0.073
Smoking, *n* (%)	42 (40.8)	53 (33.8)	0.250
Alcohol consumption, *n* (%)	31 (30.1)	45 (28.7)	0.804
Mitral isthmus block achieved, *n* (%)	92 (89.3)	138 (87.9)	0.726
Cavotricuspid isthmus block achieved, *n* (%)	100 (97.1)	152 (96.8)	0.901
Bottom line, *n* (%)	90 (87.4)	147 (93.6)	0.083
Left atrial CFAE, *n* (%)	75 (72.8)	147 (93.6)	<0.001
Right atrial CFAE, *n* (%)	54 (52.4)	141 (89.8)	<0.001
Superior vena cava, *n* (%)	19 (18.4)	23 (14.6)	0.417
Coronary sinus, *n* (%)	45 (43.7)	80 (51.0)	0.252
Ethanol ablation of the vein of Marshall, *n* (%)	14 (13.6)	23 (14.6)	0.812
Switched to AFL, *n* (%)	76(73.8)	14(8.9)	<0.001

AF, atrial fibrillation; LVEF, left ventricular ejection fractions; BMI, body mass index; PVI, pulmonary vein isolation; CFAE, complex fractionated atrial electrograms; AFL, atrial flutter.

All enrolled patients underwent PVI, roofline, MI and CTI ablation, where the block rate were 100%, 100%, 88.5% and 96.9% respectively. We completed MI bidirectional conduction block in 92 (89.3%) for termination group and 138 (87.9%) patients for CV group. The CTI was blocked in 100 (97.1%) patients in the termination group, and 152 (96.8%) patients achieved bidirectional conduction block in the CV group. In the termination group, bottom-line ablation was performed in 87.4% of patients, left atrial CFAE ablation was performed in 72.8%, and right atrial CFAE ablation was performed in 52.4%. EIVOM was done in 14 (13.6%) patients in the termination group and 23 (14.6%) in the CV group.

After a 12-month follow-up period, arrhythmia recurrence was confirmed in 45 patients (17.3%). The recurrent rate was 14.6% (15 of 103) in the termination group and 19.1% (30 of 157) in the CV group; this difference was not statistically significant (Table 3). The most common type of recurrent arrhythmia was AFL, observed in 34 of the 45 patients (75.6%) with recurrence. Among the patients with recurrence, 21 underwent a second ablation procedure. Mapping during the repeat procedure identified the recurrent arrhythmia as AF in 4 patients (19.0%), CTI-dependent AFL in 2 (9.5%), MI-dependent AFL in 10 (47.6%), roofline-dependent AFL in 2 (9.5%), and other forms of AFL in 3 (14.3%). A significant reduction in mean LAD was observed at the 1-year follow-up compared to baseline (39.57 mm vs. 42.82 mm; *P* < 0.001). *post hoc* analysis showed LA diameter were decreased in both termination and CV group (*P* < 0.001 for all).

**Table 3:** The outcome of patients.

Variables	All	Termination	CV	*P* Value
	*n* = 260	*n* = 103	*n* = 157	
Free from AF, *n* (%)	215 (82.7)	88 (85.4)	127 (80.9)	0.343
Recurrence with AFL, *n* (%)	34 (75.6)	12 (80.0)	22 (73.3)	0.624
Recurrence with paroxysmal AF, *n* (%)	7 (15.6)	2 (13.3)	5 (16.7)	0.771
Recurrence with persistent AF, *n* (%)	4 (8.9)	1 (6.7)	3 (10.0)	0.711
Repeat ablation, *n* (%)	21 (46.7)	9 (60.0)	12 (40.0)	0.205
AF	4 (19.1)	1 (11.1)	3 (25.0)	0.422
Cavotricuspid isthmus AFL	2 (9.5)	1 (11.1)	1 (8.3)	0.830
Mitral isthmus AFL	10 (47.6)	4 (44.4)	6 (50.0)	0.801
Roofline AFL	2 (9.5)	1 (11.1)	1 (8.3)	0.830
Others AFL	3 (14.3)	2 (22.2)	1 (8.3)	0.368
Left atrial diameter before RFCA, mm	42.8 ± 4.2	41.6 ± 3.9	43.6 ± 4.2	<0.001
Left atrial diameter after RFCA 1 year, mm	39.6 ± 4.1*	38.2 ± 3.7*	40.5 ± 4.1*	<0.001

AF, atrial fibrillation; AFL, atrial flutter.

Univariable and multivariable Cox regression analyses were performed to identify predictors of arrhythmia-free survival (Table 4). In the multivariable analysis, AF termination by ablation alone was not significantly associated with arrhythmia-free survival (Uni: HR: 0.739, 95% CI: 0.397–1.373, *P* = 0.754; adjusted HR: 0.765, 95% CI: 0.410–1.428, *P* = 0.400). However, intraprocedural conversion to AFL was identified as an independent protective factor against recurrence (Uni: HR: 0.319, 95% CI: 0.142–0.714, *P* = 0.005; adjusted HR: 0.306, 95% CI: 0.133–0.704, *P* = 0.005). Further analysis revealed that among patients whose arrhythmia terminated during ablation, those who first converted to AFL had a significantly lower risk of recurrence compared to those who converted directly to sinus rhythm (Uni: HR: 0.275, 95% CI: 0.108–0.696, *P* = 0.006; adjusted HR: 0.305, 95% CI: 0.119–0.784, *P* = 0.014).

**Table 4:** Survival regression analysis of recurrence in patients received RFCA.

Variable	Univariate analysis		Multivariate analysis	
	HR (95% CI)	*P-*value	HR (95% CI)	*P-*value
Male, *n* (%)	1.57 (0.73–3.38)	0.245		
Age, years	1.01 (0.97–1.05)	0.772		
AF duration, month	1.007 (1.003–1.012)	0.002	1.007 (1.002–1.013)	0.011
Left atrial diameter, mm	1.22 (1.15–1.29)	<0.001	1.24 (1.15–1.34)	<0.001
LVEF, %	1.00 (0.91–1.09)	0.932		
BMI, Kg/m^2^	1.33 (1.15–1.54)	0.000	1.33 (1.12–1.58)	0.001
Severe mitral valve regurgitation, *n* (%)	2.18 (1.02–4.68)	0.046	2.05 (0.74–5.66)	0.169
Severe tricuspid valve regurgitation, *n* (%)	2.39 (1.24–4.63)	0.010	2.57 (0.89–5.69)	0.112
Hypertension, *n* (%)	1.55 (0.86–2.80)	0.148		
Diabetes mellitus, *n* (%)	1.08 (0.58–2.01)	0.807		
Systolic blood pressure, mmHg	1.00 (0.96–1.04)	0.929		
Diastolic blood pressure, mmHg	1.02 (0.97–1.07)	0.399		
Fasting plasma glucose, mmol/L	1.36 (1.04–1.79)	0.026	1.54 (0.97–2.13)	0.066
Smoking, *n* (%)	0.79 (0.42–1.49)	0.473		
Alcohol consumption, *n* (%)	1.67 (0.92–3.03)	0.093		
Mitral isthmus block achieved, *n* (%)	1.01 (0.40–2.57)	0.978		
Cavotricuspid isthmus block achieved, *n* (%)	0.65 (0.16–2.68)	0.550		
Left atrial CFAE, *n* (%)	1.13 (0.48–2.66)	0.784		
Right atrial CFAE, *n* (%)	1.17 (0.58–2.36)	0.664		
Superior vena cava, *n* (%)	1.12 (0.52–2.40)	0.775		
Coronary sinus, *n* (%)	1.14 (0.63–2.04)	0.665		
Ethanol ablation of the vein of Marshall, *n* (%)	0.58 (0.21–1.61)	0.295		
Switched to AFL, *n* (%)	0.32 (0.14–0.71)	0.005	0.31 (0.13–0.70)	0.005
AFL ablation termination, *n* (%)	0.28 (0.11–0.70)	0.006	0.31 (0.12–0.78)	0.014
Terminate AF, *n* (%)	0.74(0.40–1.37)	0.754		

## 4 Discussion

**Figure 1:**
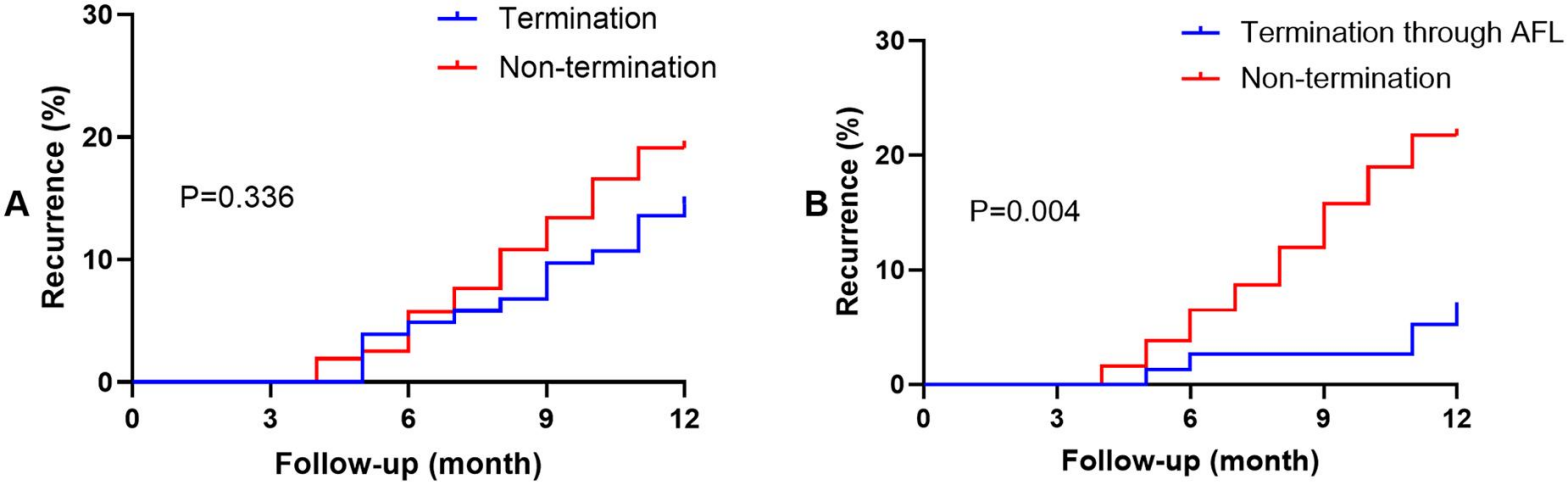
The Kaplan–Meier survival curves of AF. A, AF termination; B, AF termination with converting to AFL. AF, atrial fibrillation; AFL, Atrial flutter.

This study investigated the relationship between a comprehensive ablation strategy, termed “2C3L Plus”, procedural endpoints, and 1-year clinical outcomes in a cohort of patients with long-standing persistent atrial fibrillation (LSPAF). The primary findings reveal critical nuances in the management of this challenging arrhythmia. First, the “2C3L Plus” approach, which combines anatomical linear lesions with extensive substrate modification, achieved intraprocedural termination of atrial fibrillation (AF) to sinus rhythm without the need for electrical CV in approximately 40% of patients. This suggests that extensive ablation may effectively disrupt the mechanisms sustaining LSPAF. However, acute termination to sinus rhythm was not independently associated with a lower rate of arrhythmia recurrence at 1-year follow-up. A more subtle and potentially significant finding emerged: the intraprocedural conversion of AF to an organized AFL, followed by subsequent termination to sinus rhythm, was associated with a significantly lower recurrence rate (Figure 1). This observation suggests that the mode of termination may hold more prognostic value than termination itself.

Unlike paroxysmal AF, where ectopic triggers from the pulmonary veins are the primary drivers, LSPAF is characterized by advanced atrial remodeling. This process involves significant electrical and structural changes, including widespread fibrosis, which creates a complex and heterogeneous substrate. This remodeled tissue promotes “nonuniform anisotropic” impulse propagation, a condition where electrical signals travel at different speeds in different directions, fostering the multiple, wandering wavelets that perpetuate the disorganized rhythm of AF. Consequently, pulmonary vein isolation (PVI) alone, while the cornerstone of paroxysmal AF ablation, is often insufficient for achieving durable sinus rhythm in patients with LSPAF (6).

In response to the limitations of PVI-only strategies, various approaches targeting the atrial substrate have been developed. The “2C3L” approach is a standardized, anatomical strategy consisting of bilateral circumferential PVI (“2C”) and three predefined linear ablation lesion sets (“3L”): one across the left atrial roof, one at the mitral isthmus, and one at the cavotricuspid isthmus. This fixed protocol was developed to offer a more reproducible and efficient alternative to the earlier “stepwise” approach, which also involved linear lesions but often included time-consuming, operator-dependent mapping and ablation of CFAEs with AF termination as a primary goal. The strategy employed in the present study, “2C3L Plus”, builds upon this anatomical foundation by incorporating additional “extensive substrate ablation.” This likely involves targeted ablation of regions exhibiting CFAEs or other electrophysiological markers of abnormal substrate, creating a hybrid strategy. This is distinct from, but conceptually related to, the “upgraded 2C3L” approach, which specifically adds ethanol infusion of the vein of Marshall (EI-VOM) to improve the durability of the mitral isthmus lesion, a common site of post-ablation reentry.

The pursuit of an optimal extensive ablation strategy remains an area of active investigation and debate. Early enthusiasm for CFAE-guided ablation, which targets sites of high-frequency, fractionated electrical activity thought to be AF drivers, has been tempered by inconsistent results. There are several ablation strategies, such as box isolation, ganglionated plexi, and rotor ablation. In the STAR AF study, the CFAE + PVI ablation group had a lower incidence rate of AF recurrence than the CFAE alone group and the PVI alone group during the 1-year follow-up (7). The landmark STAR AF II trial, for instance, demonstrated that adding CFAE ablation to PVI did not improve freedom from arrhythmia in patients with persistent AF (3). Subsequent meta-analyses have largely confirmed that adjunctive CFAE ablation provides no significant incremental benefit over PVI alone, raising questions about whether these electrograms represent true arrhythmia drivers or are merely passive phenomena resulting from wavelet collisions in scarred tissue (8). This uncertainty has fueled a philosophical shift in strategy. The initial focus on functional targets (i.e., transient electrical signals like CFAEs) has evolved toward more reliable anatomical targets (i.e., creating lines of block in known reentrant pathways). The “2C3L” protocol embodies this anatomical philosophy. The “2C3L Plus” strategy investigated here represents a logical next step in this evolution, attempting to merge the reproducibility of a fixed anatomical lesion set with the individualized targeting of residual abnormal substrate. This places the current study within a broader search for a balanced approach, one that can overcome the limitations of both purely functional and purely anatomical strategies. The ongoing debate is further highlighted by trials like Alster-Lost-AF, which found no benefit to adding substrate modification to PVI (54% vs. 57% freedom from recurrence), contrasting with other studies that have shown improved outcomes with more extensive, electroanatomically guided ablation (9). Even when compared to surgical ablation, as in the CASA-AF trial which found no significant difference in recurrence rates between catheter and thoracoscopic approaches for LSPAF, it is clear that no single strategy is universally curative, reinforcing the need for refined catheter-based techniques like the one explored in this study (10). As one of “stepwise” ablation, “2C3L” was an effective approach to restore sinus rhythm. In our study, “2C3L Plus” ablation strategy was performed with CPVI, linear ablation, and left and right atrial extensive substrate ablation.

As an end point of ablation, AF termination is still controversial in terms of clinical benefit. Focal triggers, rotors and re-entry mechanisms are closely associated with the incidence and maintenance of AF. In several studies, ablation to AF termination was considered to break the AF mechanism. However, the outcome of termination was not good in some randomized trials, such as the Alster-Lost-AF and CHASE-AF trials (9, 11). In addition, AF termination was incidental and not reproducible. In our study, the termination rate was 39.6%, and there were no significant differences in outcomes between the termination group and the CV group. In addition, our study found that ablation termination was tightly linked to left atrial size and the course of AF. Several factors that might influence the clinical outcome of AF termination in extensive substrate ablation. First, recovery of conduction might play a critical role, which is a limitation of current energy ablation protocols. In recurrent patients, it is not clear if conduction is recovered or the ablation is incomplete (12). In addition, the pathogenesis of AF is progressive. The current mechanisms of AF are eliminated during ablation, which does not prevent the emergence of new mechanisms. CFAE recognition also plagues the operator, in which the ablation region is the artefact of wave collision (13). Interestingly, conversion to AFL during ablation might have promoted freedom from AF recurrence in our study. We speculate that AF conversion to AFL and termination revealed the disruption of AF maintenance more efficiently. If we complete ablation of the maintenance mechanisms rather than merely terminating the AF, the outcome might be better. The results of this study, however, suggest a different paradigm: that the organized transition from AF to AFL during ablation may be a positive prognostic indicator. This supports the hypothesis that the extensive “2C3L Plus” ablation strategy successfully disrupts the chaotic, high-frequency wavelets required to sustain AF, thereby “de-complexing” the arrhythmia into a more organized, lower-frequency AFL. This phenomenon of arrhythmia organization has been observed previously in stepwise ablation approaches, where persistent AF often converts to an organized atrial tachycardia before final termination to sinus rhythm (14).

The pattern of recurrence observed in this study further strengthens this interpretation. In the group where AF was terminated directly to sinus rhythm without an intermediate AFL phase, the predominant form of recurrence was AFL. This implies that in these patients, the ablation was sufficient to create the substrate for iatrogenic AFL (i.e., the linear lesions) but was not comprehensive enough to eliminate the core AF drivers during the index procedure. In contrast, for the group that converted to AFL intraprocedurally, the primary mechanisms sustaining AF may have been more effectively identified and eliminated, with the resulting AFL representing a simpler, residual circuit that could then be targeted and ablated.

This leads to a compelling conceptual framework where intraprocedural conversion to AFL serves as a real-time bioassay of successful substrate modification. LSPAF can be viewed as a high-entropy, disorganized electrophysiological state maintained by multiple wavelets. AFL is a more stable, lower-entropy, organized state maintained by a single macro-reentrant loop. The application of extensive ablation acts as an intervention that reduces the complexity of this dynamic system. Observing the state transition from AF to AFL provides direct, real-time evidence that the delivered energy is having the desired mechanistic effect—eliminating the substrate necessary to sustain the more complex arrhythmia. While direct termination to sinus rhythm might be a more superficial or transient phenomenon, the transition through AFL signifies a fundamental change in the arrhythmia's organizational state. Therefore, achieving this transition and then successfully ablating the resultant AFL may represent a more complete, mechanistically guided, and ultimately more durable ablation procedure. This reframes intraprocedural AFL conversion from a potential complication to a desirable intermediate endpoint, suggesting a new procedural goal: not just to terminate AF, but to first de-complex it to AFL, thereby confirming effective substrate modification, before eliminating the final organized rhythm.

Our study has some limitations. First, its retrospective, single-center design is inherently susceptible to selection bias and confounding variables that may not have been accounted for. In particular, lack of a standardized ablation process prevents a fair comparison of recurrence rates after catheter ablation. While statistical adjustments were made, this design cannot establish causality with the same certainty as a prospective, randomized controlled trial. Second, We did not perform substrate mapping prior to ablation. The uniform adoption of a 2C3L plus CFAEs ablation strategy may, to some extent, increase the incidence of iatrogenic ATs, particularly in patients with normal LA substrate. The efficacy and safety of extensive ablation beyond PVI warrant further validation in large-scale clinical studies in the future. Third, the recurrence of AF was defined by ECG, ambulatory Holter data or clinical symptoms. The use of implantable devices will improve the rigor of the evidence. Last, we found the termination of AF had the trend of lower recurrence risk but without statistical significance. This may due to underpowered statistic. Further study with large sample is needed.

## 5 Conclusion

This finding reframes intraprocedural AFL conversion from a mere procedural step into a valuable, real-time bioassay of therapeutic efficacy. Observing this transition from a chaotic, high-entropy state (AF) to an organized, lower-entropy macro-reentrant circuit (AFL) provides direct evidence that the underlying substrate has been successfully modified to the point where it can no longer sustain fibrillation.

## Data Availability

The original contributions presented in the study are included in the article/Supplementary Material, further inquiries can be directed to the corresponding author.

## References

[1] Gunawardene MA Willems S. Atrial fibrillation progression and the importance of early treatment for improving clinical outcomes. EP Europace Europace. (2022) 24:ii22–8. 10.1093/europace/euab257 35661866

[2] Sang C Liu Q Lai Y Xia S Jiang R Li S Pulmonary vein isolation with optimized linear ablation vs pulmonary vein isolation alone for persistent AF. JAMA. (2024) 333:e2424438. 10.1001/jama.2024.24438 PMC11574720 39556379

[3] Verma A Jiang C Betts TR Chen J Deisenhofer I Mantovan R Approaches to catheter ablation for persistent atrial fibrillation. N Engl J Med. (2015) 372:1812–22. 10.1056/NEJMoa1408288 25946280

[4] Dixit S Marchlinski FE Lin D Callans DJ Bala R Riley MP Randomized ablation strategies for the treatment of persistent atrial Fibrillation: RASTA study. Circ: Arrhythmia Electrophysiol. (2012) 5:287–94. 10.1161/CIRCEP.111.966226 22139886

[5] Kochhäuser S Jiang C-Y Betts TR Chen J Deisenhofer I Mantovan R Impact of acute atrial fibrillation termination and prolongation of atrial fibrillation cycle length on the outcome of ablation of persistent atrial fibrillation: a substudy of the STAR AF II trial. Heart Rhythm Heart Rhythm. (2017) 14:476–83. 10.1016/j.hrthm.2016.12.033 28011328

[6] Boyle PM del Álamo JC Akoum N. Fibrosis, atrial fibrillation and stroke: clinical updates and emerging mechanistic models. Heart Heart. (2021) 107:99–105. 10.1136/heartjnl-2020-317455 33097562

[7] Verma A Mantovan R Macle L De Martino G Chen J Morillo CA Substrate and trigger ablation for reduction of atrial fibrillation (STAR AF): a randomized, multicentre, international trial. Eur Heart J. (2010) 31:1344–56. 10.1093/eurheartj/ehq041 20215126 PMC2878965

[8] Providência R Lambiase PD Srinivasan N Ganesh Babu G Bronis K Ahsan S Is there still a role for Complex fractionated atrial electrogram ablation in addition to pulmonary vein isolation in patients with paroxysmal and persistent atrial fibrillation? Circ. Arrhythmia Electrophysiol Circ Arrhythm Electrophysiol. (2015) 8:1017–29. 10.1161/CIRCEP.115.003019 26082515

[9] Fink T Schlüter M Heeger C-H Lemes C Maurer T Reissmann B Stand-Alone pulmonary vein isolation versus pulmonary vein isolation with additional substrate modification as Index ablation procedures in patients with persistent and long-standing persistent atrial fibrillation. Circ: Arrhythmia Electrophysiol. (2017) 10:e005114. 10.1161/CIRCEP.117.005114 28687670

[10] Haldar S Khan HR Boyalla V Kralj-Hans I Jones S Lord J Catheter ablation vs. thoracoscopic surgical ablation in long-standing persistent atrial fibrillation: cASA-AF randomized controlled trial. Eur Heart J. (2020) 41:4471–80. 10.1093/eurheartj 32860414 PMC7767634

[11] Vogler J Willems S Sultan A Schreiber D Lüker J Servatius H Pulmonary vein isolation versus defragmentation: the CHASE-AF clinical trial. J Am Coll Cardiol. (2015) 66:2743–52. 10.1016/j.jacc.2015.09.088 26700836

[12] Riley MP Marchlinski FE. Termination of persistent atrial fibrillation during ablation: finding the needle in the haystack. Cardiovasc Electrophysiol. (2013) 24:1101–3. 10.1111/jce.12230 24015788

[13] Latchamsetty R Oral H. Is ablation to termination the best strategy for ablation of persistent atrial fibrillation? Circ. Arrhythmia Electrophysiol. (2015) 8:972–80. 10.1161/CIRCEP.115.001722 26286306

[14] Elayi CS Di Biase L Barrett C Ching CK al Aly M Lucciola M Atrial fibrillation termination as a procedural endpoint during ablation in long-standing persistent atrial fibrillation. Heart Rhythm Heart Rhythm. (2010) 7:1216–23. 10.1016/j.hrthm.2010.01.038 20206323

